# Measuring the Refractive Index of Highly Crystalline Monolayer MoS_2_ with High Confidence

**DOI:** 10.1038/srep08440

**Published:** 2015-02-13

**Authors:** Hui Zhang, Yaoguang Ma, Yi Wan, Xin Rong, Ziang Xie, Wei Wang, Lun Dai

**Affiliations:** 1 State Key Lab for Mesoscopic Physics and School of Physics, Peking University, Beijing 100871, China; 2 Collaborative Innovation Center of Quantum Matter, Beijing 100871, China

## Abstract

Monolayer molybdenum disulphide (MoS_2_) has attracted much attention, due to its attractive properties, such as two-dimensional properties, direct bandgap, valley-selective circular dichroism, and valley Hall effect. However, some of its fundamental physical parameters, *e.g.* refractive index, have not been studied in detail because of measurement difficulties. In this work, we have synthesized highly crystalline monolayer MoS_2_ on SiO_2_/Si substrates via chemical vapor deposition (CVD) method and devised a method to measure their optical contrast spectra. Using these contrast spectra, we extracted the complex refractive index spectrum of monolayer MoS_2_ in the wavelength range of 400 nm to 750 nm. We have analyzed the pronounced difference between the obtained complex refractive index spectrum and that of bulk MoS_2_. The method presented here is effective for two-dimensional materials of small size. Furthermore, we have calculated the color contour plots of the contrast as a function of both SiO_2_ thickness and incident light wavelength for monolayer MoS_2_ using the obtained refractive index spectrum. These plots are useful for both fundamental study and device application.

In recent years, the discovery of various kinds of two-dimensional (2D) materials^1^,^2^,^3^,^4^ has promoted low-dimensional physics research. Among these materials, monolayer MoS_2_, a stable atomically thin structure with honeycomb lattice, has attracted much attention, because of its remarkable physical properties and novel applications, such as direct bandgap^2^, strong spin-orbit coupling^5^,^6^, valley-selective circular dichroism^7^,^8^,^9^,^10^, valley Hall effect^11^,^12^, nonlinear optical effect^13^, and two-dimensional heterostructures^14^,^15^,^16^,^17^. Recent progress in the synthesis of highly crystalline monolayer MoS_2_ with chemical vapor deposition (CVD) method^18^,^19^,^20^ has made it a promising candidate for novel electronic and optoelectronic devices. Its complex refractive index in visible range is important, because many of its novel properties are closely related to this wavelength range. Beal *et al.* measured the complex refractive index of bulk MoS_2_^21^ in 1979. However, physical properties of a 2D material are usually very different from those of bulk material, especially for MoS_2_, which has an indirect bandgap in bulk form and a direct bandgap in monolayer form^2^. Recently several groups have measured the optical constants of large-area CVD grown thin films of MoS_2_ or other transition metal dichalcogenides using spectroscopic ellipsometry technique^22^,^23^,^24^. However, so far, it is still difficult to measure the refractive index of highly crystalline monolayer MoS_2_ directly, because the highly crystalline monolayer MoS_2_ flakes obtained by present methods (mechanical exfoliation, CVD *etc.*), are usually too small in size (~ tens of microns). In 2007, Blake *et al.* successfully visualized graphene under an optical microscope by utilizing the contrast of graphene on a SiO_2_/Si substrate. Given the refractive index of graphene, the contrast can further be calculated based on the Fresnel law^25^. In our work, by improving the spatial resolution of a reflectance spectrum system via spatial filtering, we managed to obtain the optical contrast spectra of highly crystalline monolayer MoS_2_. Then by curve fitting the relations between the contrast and SiO_2_ thickness based on the Fresnel law, we obtained the complex refractive index spectrum of monolayer MoS_2_ in the wavelength range of 400 nm to 750 nm, together with the confidence interval, which reflects the accuracy of the obtained refractive index. We have analyzed the pronounced difference between the obtained complex refractive index spectrum and that of bulk MoS_2_. Furthermore, we have calculated the color contour plots of the contrast as a function of both SiO_2_ thickness and incident light wavelength for monolayer MoS_2_ using the obtained refractive index spectrum. These plots are useful for both fundamental study and device application.

## Results

### Synthesis and characterization of highly crystalline monolayer MoS_2_

**Figure 1:**
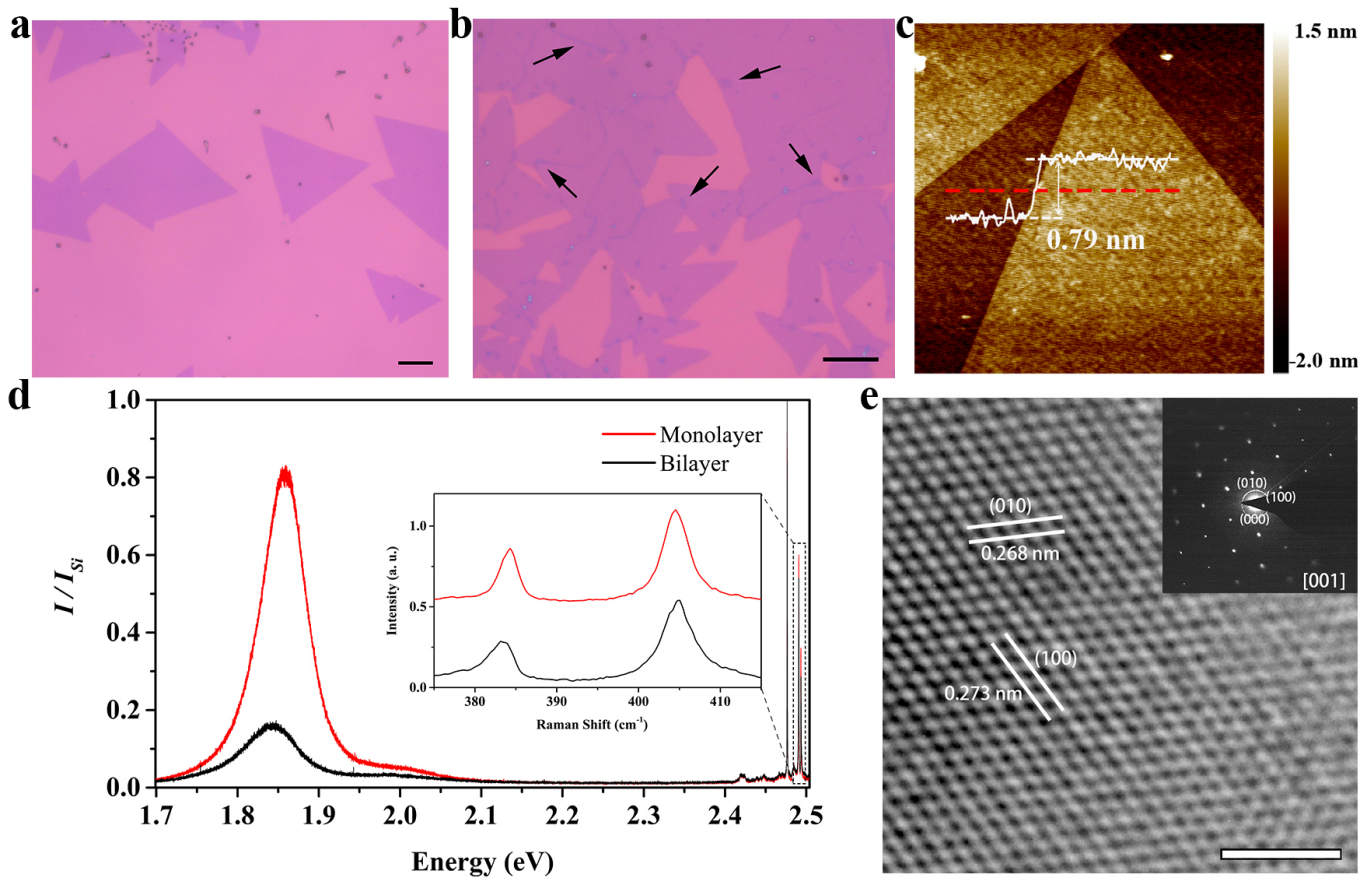
Synthesis and characterization of monolayer MoS_2_. (a) and (b) Optical images of typical MoS_2_ on 280 nm SiO_2_/Si substrates synthesized by CVD method. The growth durations are 10 min and 20 min, respectively. In (b), some monolayer MoS_2_ islands overlapped with one another because of the longer growth time, as indicated with arrows. The scale bars correspond to 10 μm. (c) AFM image of two neighbored MoS_2_ triangular islands. The white curve shows the thickness along the red dashed line. The scale bar corresponds to 0.5 μm. (d) Room-temperature PL spectra from monolayer MoS_2_ (red) and bilayer MoS_2_ (black). Peak height is normalized to the silicon Raman peak. Raman signals also appear in the PL spectra at the higher energy, details of which are shown in the inset. (e) HRTEM image of freely suspended monolayer MoS_2_. The inset is the corresponding SAED pattern recorded along the [001] zone axis. The scale bar corresponds to 2 nm.

In this work, we synthesized monolayer MoS_2_ on various SiO_2_/Si substrates by CVD method. Figures 1a, b show two typical optical images of the material grown on 280 nm SiO_2_/Si substrates. The monolayer MoS_2_ tend to form isolated triangular islands, with grain sizes ranging from a few microns to tens of microns. With increased growth time, some monolayer MoS_2_ islands overlap with one another to form bilayer or multi-layer structures as indicated with the arrows in Fig. 1b. The thickness of a MoS_2_ triangular island depicted in Fig. 1c, measured by an atomic force microscope (AFM), is about 0.79 nm, consistent with data from literatures^18^,^26^.

The photoluminescence (PL) spectra of both monolayer and bilayer MoS_2_ are shown in Figure 1d. Raman signals also appear in the PL spectra at higher energy, details of which are shown in the inset. For monolayer MoS_2_, the PL spectrum is dominated by two peaks around 1.85 eV and 2.00 eV, which come from the A and B exciton transitions, respectively. The A and B excitons form at the K-point of the Brillouin zone, where strong spin-orbit coupling induces a splitting in the opposite spin valence bands by about 150 meV^2^,^10^,^27^. For bilayer MoS_2_, the corresponding two exciton transition peaks are much weaker and exhibit a small red shift^27^,^28^. The Raman spectrum of monolayer MoS_2_ consists of the 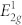 (384.3 cm^−1^) and *A_1g_* (404.6 cm^−1^) modes. Peak distance (*Δ*) of them is about 20.3 cm^−1^. The 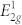 and *A_1g_* Raman modes of bilayer MoS_2_ peak at 382.9 cm^−1^ and 404.8 cm^−1^ respectively, with a *Δ* of about 21.9 cm^−1^. Peak distance between these two modes has been used to identify the number of layers of MoS_2_^29^. For monolayer and bilayer MoS_2_, the *Δ* values are about 18~20 cm^−1^ and 21~22 cm^−1^, respectively^19^,^29^. Our results are consistent with these criteria.

We characterized crystal structure of the monolayer MoS_2_ with a high-resolution transmission electron microscope (HRTEM), as shown in Fig. 1e. We can see clearly the {100} planes of MoS_2_, with a lattice spacing of about 0.270 nm^18^. The HRTEM image, together with the corresponding selected area electron diffraction (SAED) shown in the inset, demonstrates that the monolayer MoS_2_ is a single crystal with a hexagonal lattice structure.

### Measuring the contrast spectra of monolayer MoS_2_ on SiO_2_/Si substrates

**Figure 2:**
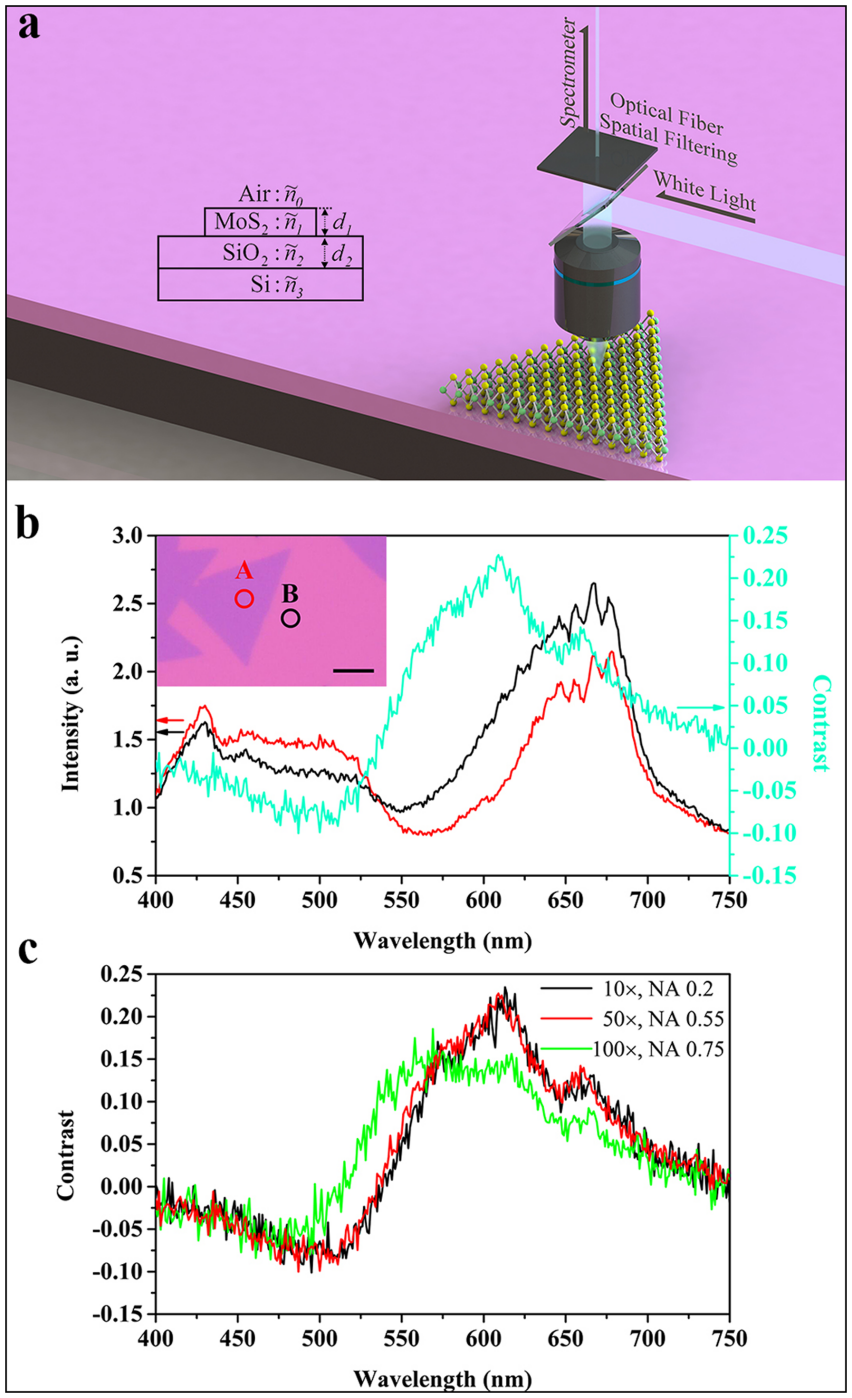
Measurement of Spatially Resolved Contrast Spectrum. (a) The schematic diagram of the experimental set-up for measuring the contrast spectrum of monolayer MoS_2_ on a SiO_2_/Si substrate. Inset: The geometric structure of our sample. (b) The reflectance spectra measured on monolayer MoS_2_ (red line) and 280 nm SiO_2_/Si substrate (black line), respectively. Corresponding measured sites are labeled as A and B in the inset. The scale bar corresponds to 10 μm. The contrast spectrum extracted from the reflectance spectra is shown by the cyan line. (c) The contrast spectra of monolayer MoS_2_ on a 281 nm SiO_2_/Si substrate collected by using three objective lenses with different N. A. (0.2 (10×), 0.55 (50×), and 0.75 (100×)).

The schematic diagram of the experimental set-up for measuring the contrast spectrum of monolayer MoS_2_ on a SiO_2_/Si substrate is shown in Fig. 2a. An optical microscope equipped with a white light source was used to collect the reflected light signal from the sample. An optical fiber was used to selectively couple part of the reflected light signal into a spectrometer. In our case, the spatial resolution of the reflectance spectrum, which depends on both the objective lens magnification and the fiber diameter, is about 1 μm under 50× objective lens, smaller than the size of the monolayer MoS_2_ islands. Figure 2b shows the reflectance spectra measured on monolayer MoS_2_ grown on a 280 nm SiO_2_/Si substrate and a bare 280 nm SiO_2_/Si substrate respectively, for wavelengths ranging from 400 nm to 750 nm. Corresponding measured sites are labeled as A and B in the inset. The contrast of monolayer MoS_2_ on a SiO_2_/Si substrate can be defined as:
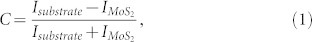
where 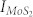 and *I_substrate_* are the reflected light intensities from MoS_2_ and the substrate, respectively. From the two reflectance spectra, we can calculate the contrast spectrum, which is also shown in Fig. 2b.

We assume normal incidence in the analysis of this work. We need to select a proper objective lens in order to minimize the error caused by the spatial Fourier components of the beam away from normal incidence^30^. On one hand, this requires the numerical aperture (N. A.) to be as small as possible. On the other hand, the lens with a smaller N. A. usually has smaller magnification, which in return reduces spatial resolution. Figures 2c shows three contrast spectra of monolayer MoS_2_ on a 280 nm SiO_2_/Si substrate, which were obtained by using objective lenses with N. A. of 0.2 (10×), 0.55 (50×), and 0.75 (100×), respectively. We can see that, apart from the spectrum collected by the lens with N. A. of 0.75, the contrast spectra collected by the other two lenses are similar, which means that the error caused by the beam away from normal incidence can be ignored when the N. A. is less than 0.55. Therefore, we used the 50× objective lens in our experiment to achieve optimal results.

### Fitting the complex refractive index of monolayer MoS_2_

**Figure 3:**
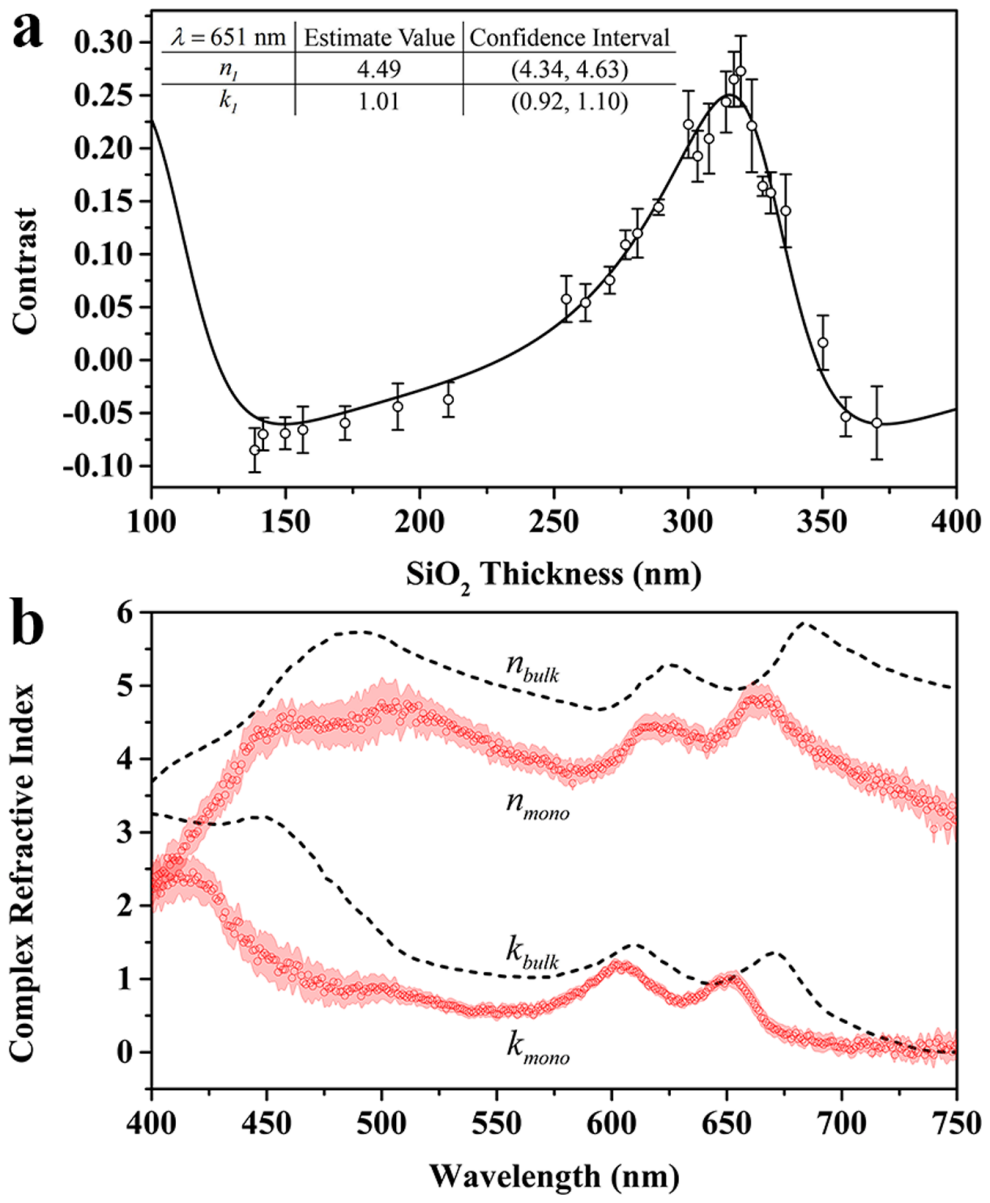
Curve Fitting results of complex refractive index for monolayer MoS_2_. (a) The contrast of monolayer MoS_2_ as a function of the SiO_2_ thickness under incident light wavelength of 651 nm (scattered open circles). The standard deviation of each point is shown by the error bar. The curve fitting result of the complex refractive index of monolayer MoS_2_ at 651 nm is shown in the table in the inset. (b)The complex refractive index spectrum (scattered red circles) of monolayer MoS_2_, obtained by curving fitting the relation between contrast and SiO_2_ thickness relation with equation (3) under different incident light wavelength (from 400 nm to 750 nm). The red shadow shows the confidence interval, corresponding to a 95% confidence level. The black dashed lines show the complex refractive index spectrum of bulk MoS_2_^21^.

For normal light incidence, the reflected light intensity from a sample with the geometric structure shown in the inset of Fig. 2a can be written as^25^: 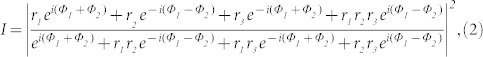
where
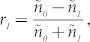

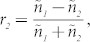

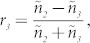
are the relative indices of refraction. 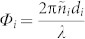 is the phase shift due to the variation of the optical path, in which *λ* is the wavelength, *d_i_* is thickness of related layer, and 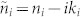 is the complex refractive index of related material. The reflected light intensity from bare SiO_2_/Si substrate can be obtained by setting 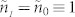. Given the theoretical thickness for monolayer MoS_2_ (*d_1_* = 0.63 nm^31^), the incident light wavelength, and the complex refractive indices of both SiO_2_ and Si, we can obtain, from equations (1) and (2), the contrast of monolayer MoS_2_ on a SiO_2_/Si substrate as a function of both SiO_2_ thickness (*d_2_*) and complex refractive index of monolayer MoS_2_ (*n_1_,k_1_*):
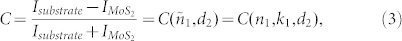
In order to obtain the complex refractive index spectrum of monolayer MoS_2_, we measured contrast spectra of 26 monolayer MoS_2_ samples on SiO_2_/Si substrates with SiO_2_ thickness ranging from ~130 nm to ~370 nm (Supplementary Fig. S1). From these contrast spectra, we extracted the relation between the contrast (*C*) and SiO_2_ thickness (*d_2_*) under a specific incident light wavelength, *e.g.* 651 nm, as shown in Fig. 3a (scattered open circles). The standard deviation of each point, indicated by its error bar, was obtained by multiple measurements. By curve fitting these data with equation (3) (the solid line), we obtained the complex refractive index of monolayer MoS_2_ (*n_1_,k_1_*) at 651 nm. Similarly, we can obtain the *C-d_2_* relations under different incident light wavelength (from 400 nm to 750 nm). The resulting complex refractive index spectrum of monolayer MoS_2_ is shown in Fig. 3b (scattered red circles), together with the confidence interval (red shadow), which corresponds to a 95% confidence level. The overall confidence interval is narrow, especially in the wavelength range from 550 nm to 700 nm, indicating that the result is with high confidence. We expect uniformity in sample properties, such as MoS_2_ thickness, crystalline quality, and surface flatness, to contribute to the confidence interval. Therefore the narrow confidence interval in the wavelength range from 550 nm to 700 nm indicates good sample uniformity, which can be attributed to the stable double-temperature-zone CVD synthesis method employed in this work as well as the fundamental thickness uniformity of the layered 2D material (the thickness can only be one, two, or more mono-layers). The observed gradually broadened confidence interval at wavelengths below 450 nm and above 700 nm may result mainly from the poor signal-noise ratio of the measuring system in those wavelength ranges. The broadened confidence interval at the wavelength range of 450–525 nm may result from the poor signal-noise ratio of the measured optical contrast, which is related to the SiO_2_ thickness employed. We can see in Fig. S1 that the absolute optical contrast of most of our samples are weaker in the wavelength range of 450–525 nm.

For comparison, we also plotted the complex refractive index spectrum of bulk MoS_2_^21^ in this figure with the black dashed lines. We can see that, for most wavelengths, the real and imaginary parts of the refractive index of monolayer MoS_2_ are both lower than their bulk counterparts. In addition, for monolayer MoS_2_, two peaks of the real part of the refractive index spectrum, located at 663 nm and 621 nm, exhibit a blue shift compared to their bulk counterparts (at 684 nm and 626 nm, respectively). Similarly, two peaks of the imaginary part (at 651 nm and 603 nm) also show a blue shift compared to their bulk counterparts (at 671 nm and 611 nm, respectively). Given that the imaginary part represents the electromagnetic wave absorption in a material, we attribute the two peaks of the imaginary part of the refractive index spectra to A and B exciton absorptions, respectively. The observed blue shifts of exciton absorption of monolayer MoS_2_ compared to their bulk counterparts may be due to the difference of A and B excitons energy in bulk and monolayer MoS_2_^2^,^27^. It is worth noting that, compared to the exciton emission peaks (located at 1.85 eV and 2.00 eV) shown in Fig. 1d, the observed A and B exciton absorption peaks of monolayer MoS_2_ (located at 651 nm and 603 nm, *i.e.* 1.90 eV and 2.06 eV) also show a blue shift. This phenomenon results from the well-known Stokes shift between absorption and emission^32^. There also exists a peak near 400 nm for the imaginary part spectrum of monolayer MoS_2_, which may be related to the convoluted C and D excitons^28^,^33^. The reason why the peak located around 480 nm of the real part of refractive index spectrum for the bulk MoS_2_ splits into two peaks for monolayer case is still unknown, and needs further study.

**Figure 4:**
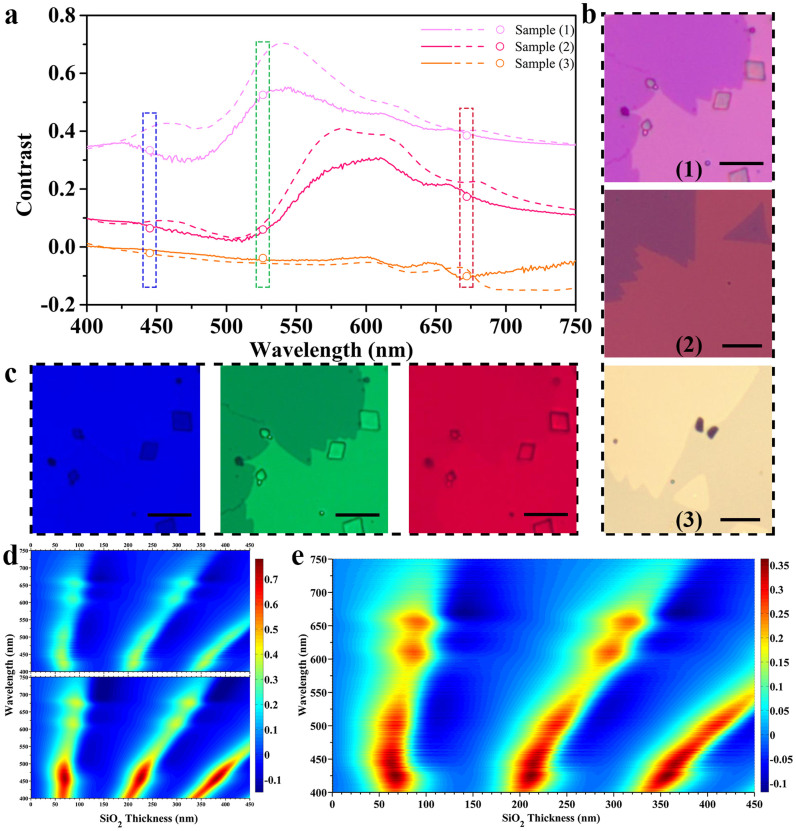
Comparison of the optical contrasts of monolayer MoS_2_ on SiO_2_/Si substrates calculated with the refractive index of monolayer MoS_2_ and that of bulk MoS_2_. (a) Three representative contrast spectra of monolayer MoS_2_ on different substrates, calculated with the refractive index of monolayer MoS_2_ (solid lines), together with those calculated with the refractive index of bulk MoS_2_ (dashed lines). The samples (1)–(3) correspond to SiO_2_ thicknesses of 262, 281, and 150 nm, respectively. The scattered open circles are the contrast data obtained directly from the monochromatic images of the three samples. (b) The optical images of samples (1)–(3). (c) The monochromic (blue, green, and red) images of sample (1) under 445 nm, 526 nm, and 672 nm light illumination, respectively. (d) The upper and lower ones are the color contour plots of the contrast as a function of both SiO_2_ thickness and incident light wavelength calculated using the refractive index of monolayer MoS_2_ and that of bulk MoS_2_, respectively. (e) The redrawing of the upper one in (d) using a different color bar. All the scale bars in Fig. 4 correspond to 10 μm.

In order to demonstrate the difference of the refractive indices for monolayer and bulk MoS_2_ from another point of view, we calculated three representative contrast spectra of monolayer MoS_2_ on SiO_2_/Si substrates, with SiO_2_ thicknesses of 262, 281, and 150 nm, respectively, labeled as samples (1)–(3). The results are plotted (solid lines) together with those calculated using the refractive index of bulk MoS_2_ (dashed lines) in Fig. 4a. Figure 4b shows the optical images of samples (1)–(3), respectively. The optical images show different colors as a result of the different reflectance spectra of the three samples. We also took the monochromatic images of the samples by inserting narrow-band filters into the illuminating optical path. As an example, three monochromatic images of sample (1) are shown in Fig. 4c. The images show quite different contrast, the blue one having the worst contrast, and the green one having the best. From the converted grayscale images, we obtained the contrast data directly. The obtained contrast data for all the three samples are plotted in Fig. 4a (scattered open circles). We can see that the measured contrast data agree well with those calculated with the refractive index of monolayer MoS_2_ (solid lines).

Using refractive indices of monolayer and bulk MoS_2_, we calculated the color contour plots of the contrast as a function of both SiO_2_ thickness and incident light wavelength for monolayer MoS_2_ on SiO_2_/Si substrate. The results were plotted in Fig. 4d under the same color bar. The clear difference between these two plots confirms again that the difference between the refractive indices of monolayer and bulk MoS_2_ cannot be ignored. For both fundamental study and device application, it is useful to make the sample visible under an optical microscope. It is common to use 280 nm–300 nm SiO_2_/Si substrates for monolayer MoS_2_ in order to visualize it. In principle, with this color contour plot, one can make the monolayer MoS_2_ visible on any SiO_2_/Si substrate by selecting the proper incident light wavelength, and vice versa. For convenient to use, we replotted the color contour plot in Fig. 4e using a more appropriate color bar.

## Discussion

In this work, we have synthesized highly crystalline monolayer MoS_2_ on 26 different SiO_2_/Si substrates with SiO_2_ thickness ranging from ~130 nm to ~370 nm, and have devised a method to measure the contrast spectra of monolayer MoS_2_ on these substrates. Using these contrast spectra, we extracted the complex refractive index spectrum of monolayer MoS_2_ in the wavelength range of 400 nm to 750 nm. We have analyzed the pronounced difference between the obtained complex refractive index spectrum and that of bulk MoS_2_. Furthermore, we have calculated the color contour plots of the contrast as a function of both SiO_2_ thickness and incident light wavelength for monolayer MoS_2_ using the obtained refractive index spectrum. These plots are useful for both fundamental study and device application. The measurement method presented here, with the advantage over conventional methods for 2D materials with small size, can be applied to other 2D materials which can be synthesized with good repeatability.

## Methods

### Preparation of SiO_2_/Si substrates

We chemically etched the thermally oxidized SiO_2_ capping layers on the Si substrates to various thicknesses (from 130 nm to 370 nm) using buffered hydrogen fluoride (HF) (HF (40%): NH_4_F (8 M) = 1:10). The original thickness of the SiO_2_ capping layer was 600 nm. The whole etching process was carried out in ice-bath with magnetic stirring. The thickness of the SiO_2_ layer was measured by a thin film thickness measurement system (SpectraThick Series, ST2000-DLXn) with repeatability of 0.5 nm. The refractive index of the *p*-Si substrate (resistivity: 8–12 Ω·cm) was measured by an ellipsometer (Horiba Jobin Yvon Uvisel) (shown in Fig. S2).

### Synthesis of monolayer MoS_2_

We synthesized monolayer MoS_2_ on SiO_2_/Si substrates with a double-temperature-zone CVD method at ambient pressure. The MoO_3_ (99.99%) and S (99.999%) powders, serving as the source, were loaded onto two quartz boats, respectively, which were later inserted into a 1-inch diameter quartz tube placed in a tubular furnace. The MoO_3_ located at the downstream of high-purity argon (Ar) carrier gas. SiO_2_/Si substrates were placed with faces down above MoO_3_. During the synthesis process, the temperatures at MoO_3_ and S sources were first raised to 100°C and kept there for 1 h with Ar gas flow rate of 120 sccm to exhaust water and air. Then the temperatures at MoO_3_ and S were ramped to 650°C and 220°C, respectively, in 40 min, and kept there for 10 min, with Ar gas flow rate of 10 sccm. After that, the furnace was cooled down to room temperature without feedback with Ar gas flow rate of 10 sccm.

### Characterization of monolayer MoS_2_

The thickness and crystal structure of monolayer MoS_2_ were characterized by AFM (Bruker Dimension Icon-PT) and TEM (Tecnai F30), respectively. For TEM sample preparation, we first spun a layer of poly (methyl methacrylate) (PMMA) onto a MoS_2_/SiO_2_/Si sample. Then we etched off the SiO_2_ layer by KOH aqueous solution (2 M) and cleaned the floating PMMA/MoS_2_ membrane several times with deionized water. Finally the membrane was scooped onto a TEM grid and dried. The PMMA was removed by annealing the TEM sample at 400°C for 3 h in an Ar/H_2_ ambient. The PL and Raman spectra of monolayer MoS_2_ were measured by a confocal Raman microscopic system (Horiba Labram HR800) at room temperature. The excitation laser wavelength was 488 nm.

### Spatially resolved spectrum system

The spatially resolved reflectance spectrum was measured by using an optical microscopic spectrum system, which included an optical microscope (Zeiss Axio Imager. A2m) equipped with a halogen lamp (Zeiss Hal 100, 12 V, and 100 W), and a spectrometer (Horiba Jobin Yvon Triax 320). One end of an optical fiber was placed at the image plane of the microscope to selectively couple part of the light signal there to the spectrometer. The diameter of the optical fiber is 9 μm.

### Obtaining Monochromatic Images

Monochromatic image was obtained by inserting a color filter with bandwidth of ~10 nm into the illuminating optical path of the optical microscope. The central wavelengths of the three filters used in Fig. 4 are 445 nm, 526 nm, and 672 nm, respectively.

## Author Contributions

L.D. and H.Z. conceived the research, analyzed the data, and wrote the manuscript. Y.M. and H.Z. devised the optical contrast spectra measurement method. H.Z. and Y.W. synthesized the samples, did the PL, Raman, and optical contrast spectra measurements. X.R. did the AFM measurement. Z.X. measured the refractive index of silicon. H.Z. did the TEM characterization and the numerical calculation (assisted by W.W.). L.D. supervised the study. All authors discussed the results.

## Supplementary Material

Supplementary Information Supplementary Information
