# Supplementary material for: Measuring the Refractive Index of Highly Crystalline Monolayer MoS2 with High Confidence
Source: Sci Rep. 2015 Feb 13;5:8440. doi: 10.1038/srep08440 (PMC4326697; doi:10.1038/srep08440)
Supplement: Supplementary Information [file srep08440-s1.pdf]

Supplementary Information

Measuring the Refractive Index of Highly Crystalline Monolayer MoS_2 with High Confidence

Hui Zhang, [1, 2] *Yaoguang Ma*, [1] *Yi Wan*, [1] *Xin Rong*, [1] *Ziang Xie*, [1] *Wei Wang*, [1] and *Lun Dai*, [1, 2, a)]

1 State Key Lab for Mesoscopic Physics and School of Physics, Peking University, Beijing 100871, China

2 Collaborative Innovation Center of Quantum Matter, Beijing 100871, China

a) To whom correspondence should be addressed. E-mail: lundai@pku.edu.cn

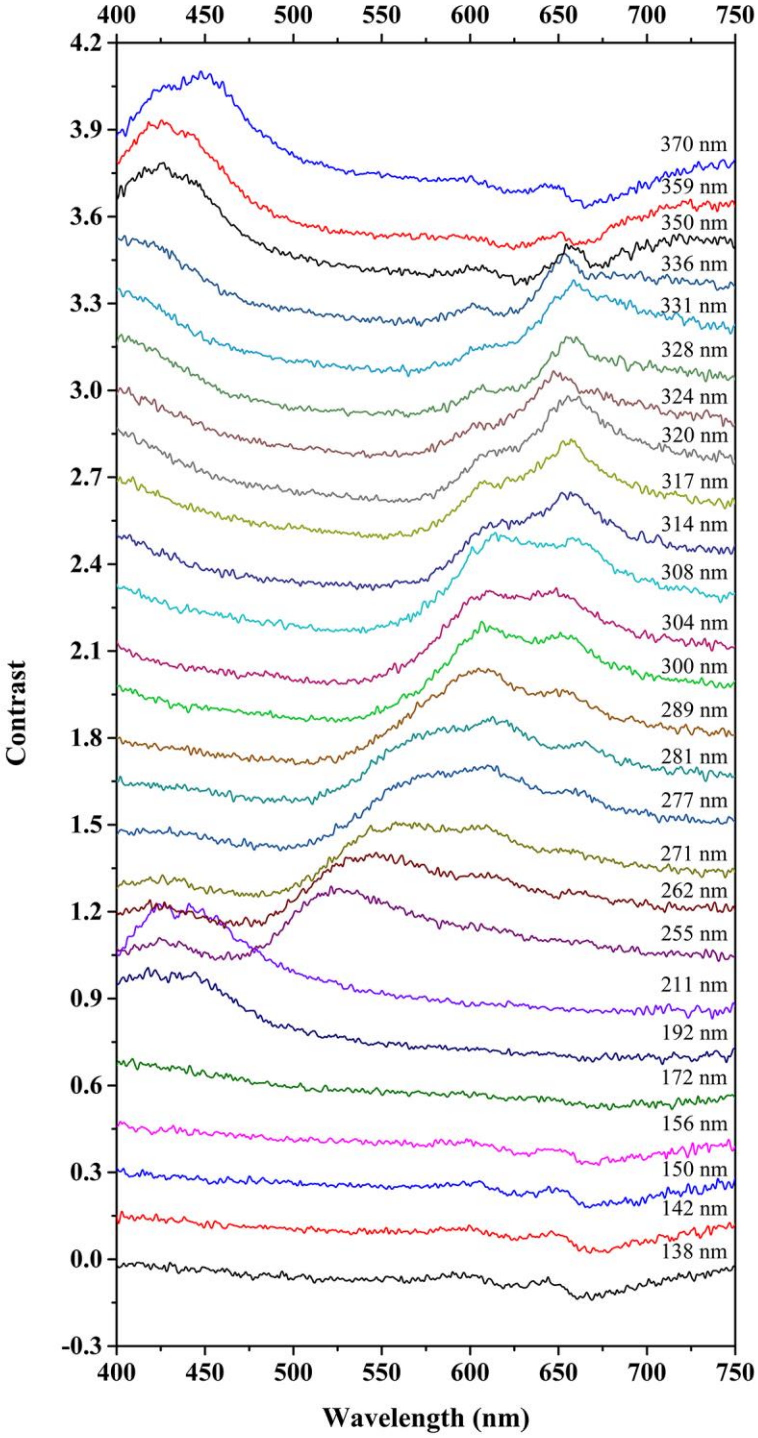

Supplementary Figure S1: The optical contrast spectra of 26 monolayer MoS_2 samples on various SiO_2/Si substrates with SiO_2 thickness changing from ~ 130 nm to ~ 370 nm. We add 0.15 to each curve in sequence to stagger them.

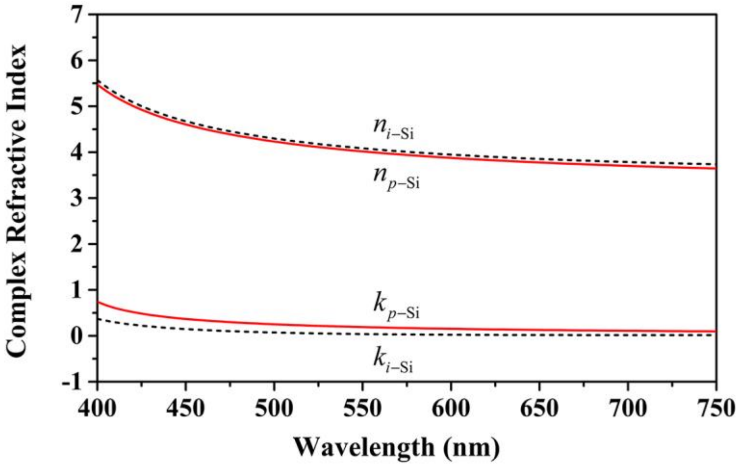

Supplementary Figure S2: The refractive index of the *p*-Si (resistivity: 8-12 Ω·cm) substrate (red solid lines) measured by the ellipsometer (Horiba Jobin Yvon Uvisel). The refractive index of intrinsic silicon[1] (black dashed lines) is also plotted in this figure for comparison.

Confidence Interval and Confidence Level:

Confidence level reflects the probability that the true value locates in the confidence interval[2]. 95% is a typical high confidence level in statistics. In our case, it means that the true value of the refractive index locates in the confidence interval with 95% probability.

The confidence interval is extracted from the estimate value of parameter, the standard error, and the t-value. The t-value is a statistic factor of Student's t-distribution[3, 4], which is determined by the confidence level and the number of samples.

Supplementary Reference:

1. Palik, E. D. *Handbook of optical constants of solids*. Academic Press (1985).
2. Cox, D. R., Hinkley, D.V. *Theoretical Statistics*. Chapman and Hall (1974)
3. Gosset, W. S. The probable error of a mean. *Biometrika* **6**, 1-25 (1908)
4. Hogg, R. V., Craig, A. T. *Introduction to Mathematical Statistics.* Macmillan (1978)
